# Health workers' use of routine health information and related factors at public health institutions in Illubabor Zone, Western Ethiopia

**DOI:** 10.1186/s12911-022-01881-y

**Published:** 2022-05-24

**Authors:** Amanuel Benti Abdisa, Kifle Woldemichael Hajito, Dawit Wolde Daka, Meskerem Seboka Ergiba, Asaye Birhanu Senay, Ketema Lemma Abdi, Muluemebet Abera Wordofa

**Affiliations:** 1 Child Development and Sponsorship Project, Jimma Town, Jimma, Ethiopia; 2 Department of Epidemiology, Faculty of Public Health, Jimma University, Jimma, Ethiopia; 3 Department of Health Policy and Management, Faculty of Public Health, Jimma University, Jimma, Ethiopia; 4 Capacity Building Mentorship Partnership Program, Faculty of Public Health, Jimma University, Jimma, Ethiopia; 5 Faculty of Public Health, Institute of Health, Jimma University, Jimma, Ethiopia

**Keywords:** Culture, Information use, Health workers, Knowledge, Perception, Skill, Ethiopia

## Abstract

**Background:**

Proper utilization of health data has paramount importance for health service management. However, it is less practiced in developing countries, including Ethiopia. Therefore, this study aimed to assess routine health information utilization and identify factors associated with it among health workers in the Illubabor zone, Western Ethiopia.

**Methods:**

A facility based cross-sectional study was conducted from March to June 2021 with a total of 423 randomly selected health workers. Data were collected using an interviewer-administered questionnaire that was developed based on the performance of routine information system management (PRISM) framework. We created composite variables for health workers' knowledge, attitude, abilities, and information utilization based on existing data. Multivariate logistic regression analysis was performed and the statistical association between the outcome and independent variables was declared using 95% CI and a *P* < 0.05.

**Results:**

About two-thirds or 279 health workers (66.0%, 95% CI 61.3, 70.4) had good health information utilization. Two-thirds of health workers think organizational decision-making culture (67.1%, 95% CI 62.6, 71.5) and facility managers' or supervisors' promotion of information use (65.5%, 95% CI 60.9, 69.9) are positive. Over half of health workers (57.0%, 95% CI 52.2, 61.6) have a positive attitude toward data management, and the majority (85.8%, 95% CI 82.2, 88.9) believe they are competent of performing routine data analysis and interpretation activities. Only about two-thirds of health workers (65.5%, 95% CI 60.9, 69.9) were proficient in data analysis and interpretation.

**Conclusions:**

The use of routine health information was lower than the national target and data from other literatures. Unacceptably large number of health personnel did not use information. As a result, efforts should be made to increase health workers' data management knowledge and skills, as well as the organizational culture of data utilization.

**Supplementary Information:**

The online version contains supplementary material available at 10.1186/s12911-022-01881-y.

## Introduction

A health information system (HIS) is one of the six building blocks of a health system that interacts with the remaining blocks. An effective HIS produces reliable and real-time evidence on the health status of a population, determinants of health and health system performance. It provides information that aids in the direction of activities in all other components of the health system, such as the health workforce, service delivery, access to essential medicines, finance, and health system leadership and governance. Beyond health system management, the routine health information system can be a sources of data for research purposes [1–8].

Proper information utilization is considered the foundation for effective health system performance and a strategy to attain health-related targets in the Sustainable Development Goal (SDG) era. Despite this fact, the narrow scope and weaknesses of the existing information systems coupled with a low culture of information use are hindering the progress made towards health goals. The major determinants of information use are categorized as technical factors, behavioral factors, and organizational factors. Low access to quality health data, influenced by a lack of data management and analysis capacity, a lack of information promotion culture at organizations, and an unfavorable attitude toward data, continue to be the main challenges to information utilization in low and middle income countries’ health system [9–14].

The government of Ethiopia has considered strengthening the health information system as a mechanism to enable effective monitoring and evaluation of health policies, programs, projects and strategies since 2006. However, until the fourth health sector development plan (HSDP IV), little emphasis has been given to evidence based decision-making. In Ethiopia, the main source of evidence for routine health decisions is the health management information system, and policy-level decisions are made based on evidence generated through national surveys, censuses and planned operational research. Following the introduction of the health sector transformation agenda, data-driven decision making has taken due consideration as it is reflected in one of the agendas, ‘Information Revolution’ [15, 16].

Though Ethiopia has implemented routine health information systems for more than a decade, the progress made in terms of information utilization is steady. Information utilization among health workers stands at 57.42% [17]. Whereas cross-sectional studies revealed that information utilization among health workers in Ethiopia ranged from 37.3 to 78.5% [18, 19]. Weak information utilization at the point of data generation is attributed to various factors, mainly grouped under technical, organizational, and behavioral factors. Health worker’s characteristics, availability of HIS focused training and supportive supervision, good perceived culture of health information, having a standard set of indicators, competence of health workers on health information tasks, and good governance were the determinants of information use [17, 19, 20]. Understanding the factors affecting of health information use at the point of data generation by taking into consideration the broader determinants has paramount importance for improving the health information system. Therefore, this study aimed to determine the level of information utilization and factors associated with it among health workers and health care managers at various levels of the health system in Illubabor zone, Oromia regional state, Ethiopia.

## Methods and materials

### Study setting and period

**Fig. 1:**
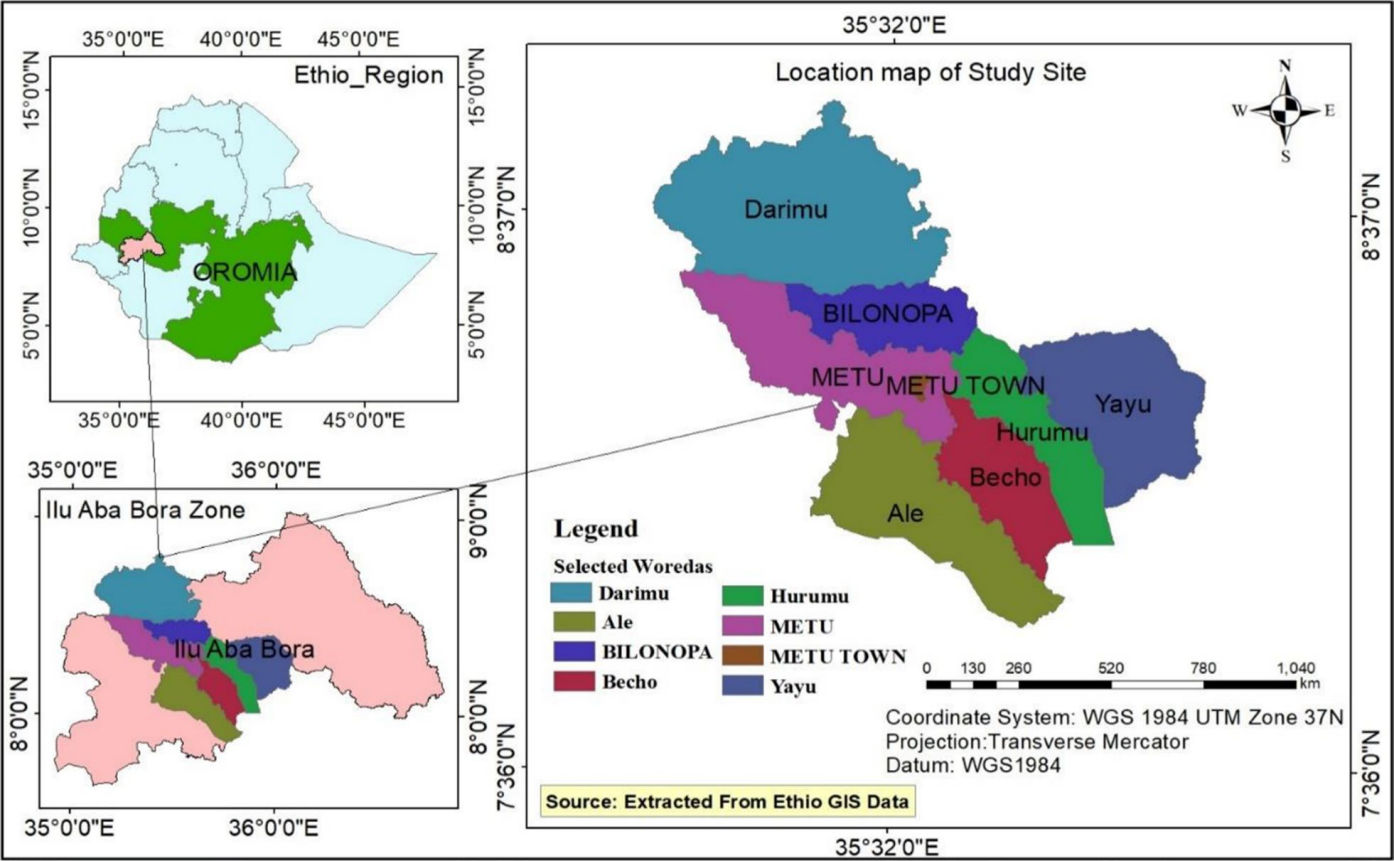
Map of the study area

The study was conducted in public health facilities and health administrations of the Illubabor zone, Western Ethiopia. Illubabor is one of the 20 zonal administrations in Oromia Regional State, located 600 km west of Addis Ababa, the capital city of Ethiopia. The total population of the zonal administration was 968, 303 as of 2020. The zonal administration is comprised of 14 rural woredas, 1 town administration, 23 urban kebeles and 263 rural kebeles. Regarding health service coverage, there were 2 hospitals, 41 health centers, and 263 health posts. Moreover, the zone was comprised of 1114 health workers with various professional categories and 606 supportive staff. Of the health workers, 60 (5.4%) were health informatics technicians (HIT) [21]. The study was conducted from March to June 2021 (Fig. 1).

### Study design and participant’s selection

An institution-based cross-sectional study was conducted in the selected health facilities and health administrations of the zone. All health workers who were directly involved in health data management (recording, data collection, data aggregation, data analysis, interpretation, and reporting), as well as those who were the focal of the department or the head of the health institution, and those with six months or more of service experience, were eligible. Health workers who were on annual leave, sick leave, maternity leave, and study leave were excluded.

The sample size was determined by using the OpenEpi Version 3.01 sample size calculator for cross-sectional surveys [22] considering the assumptions and parameters: 95% confidence level, 5% margin of error, proportion of routine health information among health professionals as 78.5% from the study conducted in Gondar [19], 1.5 design effect, and a 10% non-response rate. The calculated sample size yields 428.

A multi-stage sampling strategy was employed to select health facilities, health administrations, and study participants. In the first stage, 50% of woredas (n = 7) were selected randomly. We have included all health centers in the selected woredas and hospitals in the zone. Health Workers were selected using a purposive sampling strategy. The selected 7 woredas comprise of 23 health centers. Eight health workers that fulfilled the inclusion criteria were included in the study from each of the selected health centers (n = 184). A total of 50 health workers were selected from each of the hospitals, making a total of 100 health workers and eighteen health workers were selected from the selected woreda health offices (n = 126). Moreover, eighteen health workers were selected and included in the study from the zonal health department.

The government of Ethiopia is administratively divided into regional states and city administrations. Regions are further divided into zones and zones into lower administrative units called Woredas or districts. Whereas, Woredas are sub divided into the lowest administrative units called a kebele. Woredas serve an estimated 100,000 people and are governed by an elected Woreda council. Based on health system of Ethiopia, on average Woredas have 20 health posts, 4 health center and 1 primary hospital. Thus, the woreda health office is responsible for managing the health facilities under it. Each health units (zonal health department, woreda health office, health facilities) of Ethiopian health system have departments or case teams equipped with one focal and health workers under it responsible for coordinating and managing health service delivery [23].

### Data collection

A structured questionnaire was used to collect the data. The questionnaire was adapted from the Performance of Routine Information System Management (PRISM) framework [24]. Moreover, a template was developed to collect data regarding the availability of routine health information system inputs and visualizations in the health facilities and health administrations. The paper based questionnaire was uploaded to the Open Data Kit (ODK) for data collection. Additional file 1: The questionnaire file.

The data collection tool was translated into local languages (Afan Oromo and Amharic) and then back translated into English by two independent experienced translators to ensure consistency. A pre-test was conducted on 5% of the sample size and corrections were made.

Seven data collectors who were health professionals and had a bachelors of science degree were recruited from outside of the study area. Moreover, two supervisors with a health background and a master’s degree were involved in the study. A two-day training was given to data collectors and supervisors focused on data collection tools, data collection techniques, research ethics, and the application of the Open Data Kit (ODK). Data were collected at the health institutions after taking consent from health workers. Supervisors have closely monitored the overall data collection process and provided support at the field level whenever needed.

### Study variables

The outcome variable was routine health information utilization. The independent variables were health workers’ characteristics (age, sex, service experience, qualification, professional background and position); health workers’ training, mentorship, and supervision status related to routine health data management; health workers’ knowledge of routine health data and its management; health workers self-perception of health data and its management; perceived organizational culture of information use promotions; and health workers’ self-efficacy and skill in a data analysis and interpretation.

Routine health information utilization was measured using 10 items with a Likert scale ranging from strongly disagreed (1) to strongly agreed (5). Among the topics covered were use of data for day-to-day management of health services, identifying and managing epidemics, observing the trend of health services in the catchment area, planning, drug supply and management, disease prioritization, resource allocation, monitoring performance, decision-making, and community mobilization and discussion. Those health workers above the mean value were categorized as having good information use practices and those below the mean value as having poor information use practices.

The health worker’s knowledge was measured using 27 items, each comprised of ‘Yes/No’ responses. Health workers who responded above median value of the items were regarded as having good comprehensive knowledge, while those below the median value were categorized as having poor comprehensive knowledge.

Six items on a scale of '0' to '10' were used to assess health workers' self-efficacy in health data analysis and interpretation. The self-efficacy items are comprised of: ‘I can check data accuracy, I can calculate percentage or rate correctly, I can plot trends on a chart, I can explain the findings of data analysis and its implications, and I can use data to identify performance gaps and their root cause’. To categorize health workers' self-efficacy as "High" (> 34) or "Low" [33], a threshold demarcation formula [(Total highest Score-Total lowest score)/2] + Total lowest score was used.

Health workers’ skills towards data analysis (computation of percent and rate) and interpretation were measured using 6 items. Correct responses were labeled as ‘Yes = 1’ and incorrect responses as ‘No = 0’. The questions were distributed to health workers, and responses were collected. Health workers who scored above the median value were regarded as having ‘high competency’ and those below median value as having ‘low competency’.

Health workers’ self-perception of health data and management was measured using 6 items having a Likert scale ranging from “strongly disagree” (1) to “Strongly agree” (5). Mean value was used as a cutoff point to categorize health workers’ perception as “favorable” (Mean-value ≥ 21.0) and “unfavorable” (Mean-value < 21).

The perceived information use promotion culture of the organization was assessed using ten items on a Likert scale ranging from strongly disagree (1) to strongly agree. (5). A demarcation formula was used to categorize health workers’ perception as ‘Favorable’ (value > 31) and ‘Unfavorable’ (value < 30). Likewise, promotion of information utilization by facility managers or supervisors was measured using 10 items and categorization was made using a demarcation formula: ‘Favorable’ (value > 31) and ‘Unfavorable’ (value < 30). Information use promotion by department staff was measured using eight items ranging from strongly disagree (1) to strongly agree (5). The mean value was used as a cutoff value to categorize department staff’s promotion as ‘favorable’ and ‘unfavorable’.

### Data processing and analysis

Each data record was checked for completeness and consistency, and duplicated records were removed. The data were transferred to SPSS version 25 for analysis, and descriptive statistics were mostly utilized to describe and summarize the characteristics of health workers. Secondly, bivariate logistic regression was used to identify candidate variables for multivariate logistic regression analysis. The primary outcome of the study was routine information utilization from the health management information system. In the bivariate logistic regression variables with *P* < 0.25 were taken as candidates to multivariate logistic regression. The strength of association was expressed in Odds ratio, and 95% confidence interval and *P* < 0.05 were used as cut-off point to declare significance in the final model.

## Results

### Characteristics of health workers

A total of 423 health workers participated in the study, with a response rate of 98.8%. Approximately four out of every ten health workers (43%) came from health centers, and nearly one-third (29%) came from the woreda health office. Six out of ten of the health workers (60%) were males and most (92%) had two or more years of service experience. The mean age of health workers was 31 years (SD 5.85) with a minimum and maximum age of 20 and 57 years, respectively. In terms of profession, nurses and midwives made up the majority (55%) of health workers, followed by health officers (14%).

Three hundred seventy-five (88.7%) health workers were trained on health information system. Out of this, 193(57%) were trained before the last 12 months of survey period and 144(43%) trained in the last 12 months.

Three hundred ninety-three (92.9%) health workers had received at least one supportive supervision focused on health information system in the last six months of the survey period. Likewise, 297 (70%) of health workers were mentored at least once in the last 6 months of the survey period. Out of those health workers supervised in the last 6 months, 194(49.4%) were supervised once, 160(40.7%) were supervised twice, and 39 (9.9%) have been supervised three or more times (Table 1).

**Table 1:** Characteristics of health workers, and their training and supervision status in public health institutions of Illubabor zone, Ethiopia, March to June 2021

Variables	Frequency (N = 423)	Percent (%)
Age in years		
20–24	41	9.7
25–29	175	41.4
30–34	110	26.0
35–39	69	16.3
≥ 40	28	6.6
Work place		
Admin unit	144	34.0
Hospital	95	22.5
Health center	184	43.5
Sex		
Male	255	60.0
Female	168	40.0
Service experience in years		
≤ 5 years	111	26.2
6–9 years	181	42.8
≥ 10 years	131	31.0
Profession		
Master’s degree in public health	30	7.1
Physician	7	1.7
Health officer	59	13.9
Nurses and midwifery	234	55.3
Health informatics technician	30	7.1
Laboratory professionals	21	4.9
Druggist or pharmacist	18	4.3
Other profession^a^	24	6.1
Position or title		
Head	168	39.7
Expert	255	60.3
RHI training		
Last 12 months	144	34.0
Before last 12 months	193	45.6
No training	86	20.3
RHI supervision last 6 months		
Yes	393	92.9
No	30	7.1
RHI supervision frequency in the last 6 months (n = 393)		
Once	194	49.4
Twice	160	40.7
Three or more times	39	9.9

^a^Other profession: environmental (n = 10), health education (n = 5), health extension worker level-IV(n = 5), applied biology (n = 1), health service management (n = 1), anesthesia (n = 1), biomedical (n = 1)

### Knowledge of data management and use

**Fig. 2:**
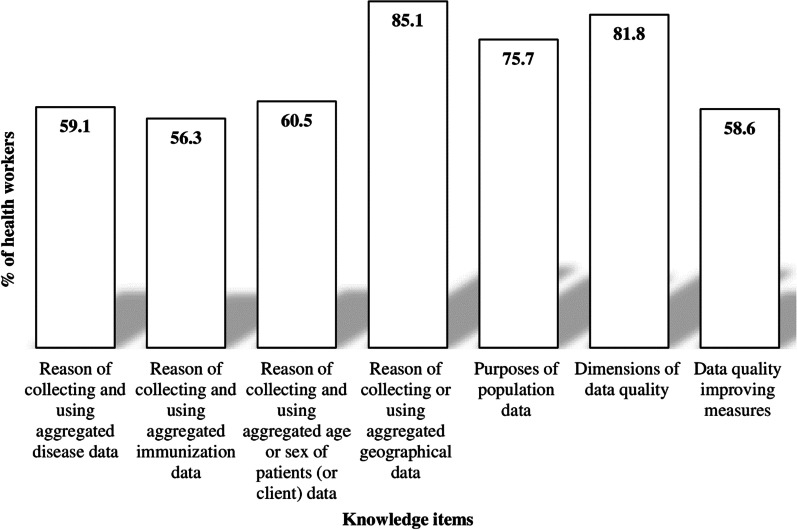
Health workers’ knowledge of data management and use

Six out of ten of the health workers had good knowledge of the reasons for collecting and using aggregated disease data (59.1%) and aggregated immunization data (62.2%). The majority of health workers (85.1%) had a strong understanding of why aggregated geographic data is collected and used, as well as the purposes of population or demographic data (75.7%). Eight out of ten health workers (81.1%) had an excellent understanding of data quality aspects, while 58.6% had a good understanding of data quality improvement measures (Fig. 2).

### Promotion of information use in the workplace (decision-making climate)

A majority of health workers agreed that organizational decisions were made based on evidence and data (72.6%), history/what was done in the previous periods (73.3%), health sector strategic objectives (74%), health needs of the catchment population (71.9%), relevant cost of interventions (71.2%), and taking inputs from relevant staff (69.7%). Overall, two-thirds (67.1%, 95% CI 62.6, 71.5) of health workers agreed that the organizational decision making climate was favorable (Table 2).

**Table 2:** Information utilization promotion culture at public health institutions of Illubabor zone, Ethiopia, March to June 2021

Variable	Frequency (N = 423)	Percent (%) (95% CI)	Mean (SD)
In organization decisions are made based on:			
Personal preference of decision makers	230	54.4(49.6–59.1)	3.0(1.07)
Superior directives	199	47.0(42.3–51.8)	3.2(1.07)
Evidence/ facts/data	307	72.6(68.2–76.7)	3.7(0.99)
History/ what was done last year	310	73.3(68.9–77.3)	3.4(0.99)
Funding directives from higher level	235	55.6(50.8–60.3)	3.0(1.22)
Political consideration	226	53.4(48.7–58.2)	2.9(1.21)
Health sector strategic objectives	313	74.0(69.7–78.0)	3.7(0.97)
Health needs of the catchment population	304	71.9(67.4–76.0)	3.6(0.95)
Relative cost of interventions	301	71.2(66.7–75.3)	3.5(0.95)
Taking inputs from relevant staffs	295	69.7(65.2–74.0)	3.5(0.97)
Organizational decision making climate is:			
Favorable	284	67.1(62.6–71.5)	
Unfavorable	139	32.9(28.5–37.5)	
Health facility managers or supervisors:			
Seek inputs from relevant staffs	301	71.2(66.7–75.3)	3.52(0.95)
Emphasis that data quality procedures be followed in the compilation and submission of period reports	289	68.3(63.8–72.6)	3.50(1.01)
Promote feedback mechanism to share or present information within the team and to lower and upper level of the system	298	70.4(66.0–74.7)	3.37(1.04)
Use routine health information system data for service performance monitoring and target setting	319	75.4(71.1–79.3)	3.67(0.88)
Emphasis the need to use RHIS data to identify potential disparities in service delivery or use	301	71.2(66.7–75.3)	3.40(1.04)
Conduct routine data quality checks at points where data are captured, processed and aggregated	299	70.7(66.2–74.9)	3.40(1.02)
Ensure that performance data are reviewed and discussed in the regular meetings	271	64.1(59.4–68.5)	3.24(1.10)
Ensure that decisions are made and follow-up actions identified in performance monitoring team meetings based on presented data	264	62.4(57.7–66.9)	3.24(1.08)
Provide regular feedback on reported data quality to the person responsible for compiling and reporting data	237	56.0(51.3–60.7)	3.05(1.11)
Recognize or reward for good work performance	208	49.2(44.4–53.9)	2.83(1.27)
Routine health information system promotion by facility managers or supervisors is:			
Favorable	277	65.5(60.9–69.9)	
Unfavorable	146	34.5(30.1–39.2)	

### Information use promotion by facility managers or supervisors

Majority of health workers agreed that health institution managers or supervisors seek inputs from relevant staffs (71.2%), emphasis data quality procedures to be followed during data management (68.3%), promote health information system feedback mechanisms (70.4%), and ensure that performance data are reviewed and discussed in the regular meetings (64.1%). Overall, two-thirds of health workers (65.5%, 95% CI 60.9, 69.9) agreed that RHIS promotion by facility managers or supervisors was favorable (Table 2).

### Promotion of information use by facility staffs

A majority of the health workers agreed that department staff in their health institutions complete RHIS tasks (69%), display commitment to ensure data quality and evidence-based decision making (68.1%), are held accountable for poor performance (74.7%) and prepare data visuals showing achievements toward targets (72.1%). Overall, seven out of ten (71.9%, 95% CI 67.4, 76.0) of health workers agreed that department staff had a favorable information use promotion culture in their health institutions (Table 3).

**Table 3:** Information use promotion by department staffs at public health institutions of Illubabor zone, Ethiopia, March to June 2021

Variable	Frequency	Percent (%) (95% CI)	Mean (SD)
Department staffs-			
Complete RHIS task (recording, reporting, processing, aggregation and reporting) on time	292	69.0(64.5–73.3)	3.52(0.99)
Display commitment to ensure data quality and evidence-based decision making	288	68.1(63.5–72.4)	3.53(0.98)
Pursue indicative national targets and set feasible local targets for essential service performance	304	71.9(67.4–76.0)	3.42(0.99)
Feel personal responsibility for failing to reach performance targets	299	70.7(66.2–74.9)	3.38(1.04)
Prepare data visuals (graphs, tables, maps) showing achievement towards targets	305	72.1(67.7–76.2)	3.59(0.92)
Can monitor whether an initiative or intervention achieved the target or goal	305	72.1(67.7–76.2)	3.33(1.06)
Are held accountable for poor performance (e.g., failure to meet reporting deadlines)	316	74.7(70.4–78.7)	3.68(0.95)
Admits mistakes (related to data management) if/when they occur and take corrective action	304	71.9(67.4–76.0)	3.61(0.96)
Promotion of information use by department staffs			
Favorable	304	71.9(67.4–76.0)	
Unfavorable	119	28.1 (24.0–32.6)	

### Health workers’ perception on RHIS data and management

**Fig. 3:**
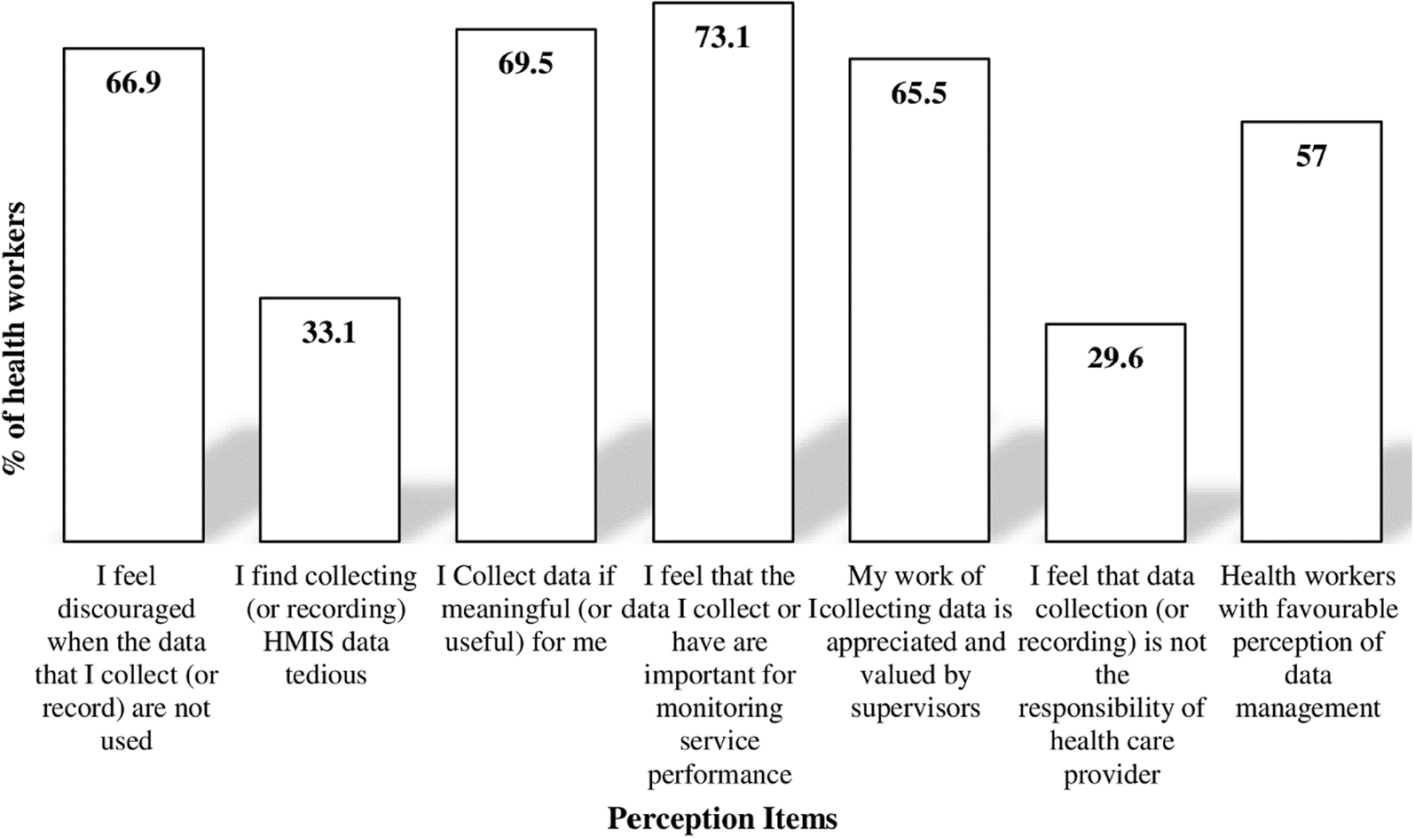
Health workers’ perception towards data management

A majority of health workers feel discouraged when data collected is not used (66.9%), and collect data only if it is useful to them (69.5%). One-thirds of health workers believe that collecting data is tedious (33.1%) and that data collection tasks are not the responsibility of healthcare providers (29.6%). Overall, over half (57.0%, 95% CI 52.2, 61.6) of the health workers had a favorable attitude towards RHIS data and its management (Fig. 3).

### Self-efficacy of data management and use

With rates ranging from 58.9 to 75.9%, the majority of health workers believe they can use data for operational decisions, interpret data analysis findings, identify performance gaps, calculate percentages and rates, plot trends on charts, and check data accuracy. Overall, greater than eight out of ten (85.8%, 95% CI 82.2, 88.9) of health workers believe that they had a high self-efficacy of data analysis, interpretation and use (Table 4).

**Table 4:** Health workers’ self-efficacy of data analysis, interpretation and use

Variable	Frequency	Percent (%) (95% CI)	Mean (SD)
I can check data accuracy	321	75.9(71.6–79.8)	7.35(1.59)
I can calculate percentage (or rate) correctly	306	72.3(67.9–76.5)	7.45(1.82)
I can plot a trend on chart	307	72.6(68.2–76.7)	7.25(1.71)
I can explain the findings of data analysis and their implications	274	64.8(60.1–69.2)	6.95(1.77)
I can use data for identifying performance gaps and its root cause	304	71.9(67.4–76.0)	7.19(1.56)
I can use data for operational (or management) decision	249	58.9(54.1–63.5)	6.66(1.72)
Health workers self-efficacy			
High	363	85.8(82.2–88.9)	
Low	60	14.2(11.1–17.8)	

### Data analysis and interpretation skill of health workers

**Fig. 4:**
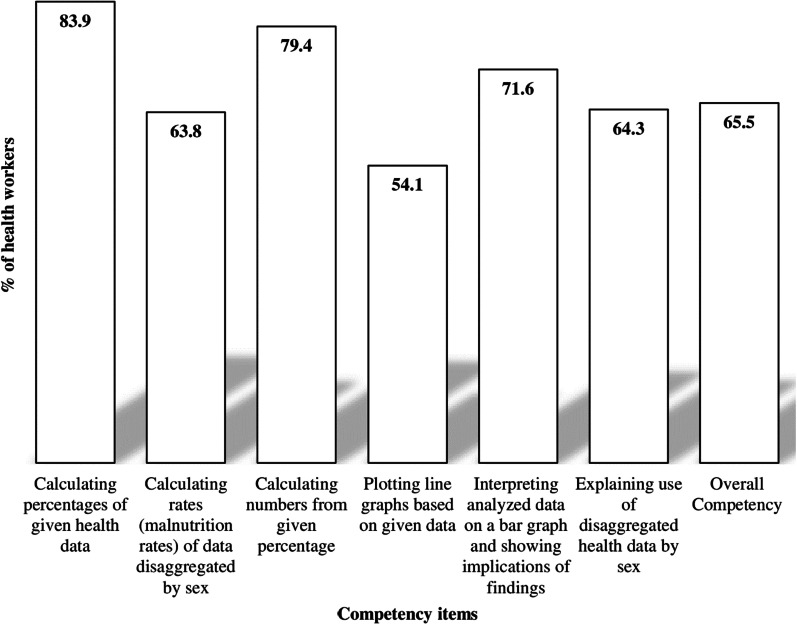
Health workers’ competency of data analysis and interpretations

The majority of health workers had high competency in data analysis (83.9%), and interpreting data (71.6%). Slightly more than half (54.1%) of health workers were competent in plotting graphs based on given data. Overall, two-thirds (65.5%, 95% CI 60.9, 69.9) of health workers had high competency in data analysis and interpretation (Fig. 4).

### Information utilization

The majority of health workers use data to identify and manage epidemics (75.7%), to observe the trend of health services in the catchment area (72.8%), to plan (81.8%), to manage medicine supply and management (77.5%), and disease prioritization (79.2%). Information was used by slightly more than half of health workers (56.3%) for day-to-day management of health services. Overall, two-thirds of health workers (66%, 95% CI 61.3, 70.4) had good information use habits (Table 5).

**Table 5:** Information use practices of health workers

Variable	Frequency	Percent (%) (95% CI)	Mean(SD)
I often use data for day-to-day management of health service	238	56.3(51.5–60.9)	3.04(1.04)
I often use data to identify and manage epidemics	320	75.7(71.4–79.6)	3.62(0.85)
I use data to observe the trends of health services in my catchment	308	72.8(68.4–76.9)	3.57(0.90)
I often use data for planning	346	81.8(77.9–85.3)	3.81(0.82)
I use data for drug supply and management	328	77.5(73.4–81.3)	3.69(0.83)
I often use data for disease prioritization	335	79.2(75.1–82.9)	3.75(0.80)
I often use data for resource allocation	320	75.7(71.4–79.6)	3.62(0.88)
I use data for monitoring performance	307	72.6(68.2–76.7)	3.61(0.87)
I use data for decision making	302	71.4(67.0–75.6)	3.55(0.93)
I often use data for community mobilization and discussion	293	69.3(64.7–73.5)	3.52(0.93)
Overall information utilization			
Good practice	279	66.0(61.3–70.4)	
Poor practice	144	34.0(29.6–38.7)	

### Factors associated with information utilization

Health workers' service experience, title or position, work place, RHI trainings, knowledge of data quality improving strategies, self-efficacy of data analysis and interpretations, data analysis and interpretation competency, perceived organizational culture of information use promotion, and information use promotion by department staffs were all statistically associated with good information use practices among health workers in the multivariate logistic regression. The following is the interpretation:

When comparing health workers with more than or equal to 10 years of service experience to those with less than 5 years, the odds of good information use practices were four times higher (AOR = 4.01, 95% CI 1.59, 10.12). When compared to experts, head health workers were twice as likely to have good information use practices (AOR = 1.85, 95% CI 1.01, 3.39).

Information use practices were 85% (AOR = 0.15, 95% CI 0.05, 0.41) and 86% (AOR = 0.14, 95% CI 0.05, 0.39) less likely among health workers who had received training on RHI compared to those who did not train. The odds of health information utilization were 58% (AOR = 0.42, 95% CI: 0.18, 0.98) less likely among health workers at hospitals compared to administrative unit (zonal or woreda health offices). The odds of information use were two times (AOR = 2.01, 95% CI 1.16, 3.47) more likely among health workers who were knowledgeable about data quality improving strategies compared to their counter parts.

Health information utilization was 2.5 times (AOR = 2.51, 95% CI 1.17, 5.36) more likely among health workers with high self-efficacy of data analysis and interpretation compared to low self-efficacy. Whereas, the odds of information utilization were three times (AOR = 2.90, 95% CI 1.71, 4.91) more likely in health workers who were competent in data analysis and interpretation compared to their counterparts.

Good information use practice was about three times (AOR = 2.61, 95% CI 1.43, 4.77) more likely among health workers who perceived organizational information use promotion culture as favorable compared to those who perceived it as unfavorable. Moreover, the odds of good information use were 2.5 times (AOR = 2.46, 95% CI 1.19–5.08) more likely among health workers who perceived the promotion of information use by department staff as favorable compared to their counterparts. The gender of health workers has only a marginal relationship with information use practices (Table 6).

**Table 6:** Factors associated with information utilization

Variables	Information Utilization		COR (95% CI)	AOR (95% CI)
	Good (n, %)	Poor (n, %)		
Age in years				
20–24	26(9.3)	15(10.4)	Ref	
25–29	117(42.0)	58(40.3)	1.16(0.57–2.37)	
30–34	67(24.0)	43(29.9)	0.90(0.43–1.89)	
≥ 35	69(24.7)	28(19.4)	1.42(0.66–3.08)	
Sex				
Male	178(63.8)	77(53.5)	1.53(1.02–2.31)**	1.67(0.91–3.04)
Female	101(36.2)	67(46.5)	Ref	Ref
Service experience in years				
** ≤ **5 years	75(26.9)	36(25.0)	Ref	Ref
6–9 years	104(37.3)	77(53.5)	0.65(0.40–1.06)*	1.55(0.72–3.32)
≥ 10 years	100(35.8)	31(21.5)	1.55(0.88–2.73)*	4.01(1.59–10.12)***
Title or position				
Head	128(45.9)	40(27.8)	2.20(1.43–3.40)**	1.85(1.01–3.39)**
Expert	151(54.1)	104(72.2)	Ref	Ref
RHI training				
Yes, last 12 months	81(29.0)	63(43.8)	0.12(0.05–0.28)**	0.15(0.05–0.41)***
Yes, before 12 months	121(43.4)	72(50.0)	0.16(0.07–0.36)**	0.14(0.05–0.39)***
No training	77(27.6)	9(6.2)	Ref	Ref
RHI supervision				
No visit	26(9.3)	4(2.8)	Ref	Ref
One visit	107(38.4)	87(60.4)	0.19(0.06–0.56)**	0.38(0.09–1.67)
Two visit	117(41.9)	43(29.9)	0.42(0.14–1.27)*	0.52(0.12–2.34)
Three or more visit	29(10.4)	10(6.9)	0.45(0.13–1.60)*	0.79(0.13–4.94)
Work place				
Admin unit	107(38.4)	37(25.7)	Ref	Ref
Hospital	40(14.3)	55(38.2)	0.25(0.15–0.44)**	0.42(0.18–0.98)**
Health center	132(47.3)	52(36.1)	0.88(0.54–1.44)	1.66(0.74–3.73)
Knowledge on reason for collecting and using aggregated disease data				
Poor knowledge	127(45.5)	46(31.9)	Ref	Ref
Good knowledge	152(54.5)	98(68.1)	0.56(0.37–0.86)**	1.21(0.65–2.26)
Knowledge on reason for collecting and using aggregated immunization data				
Poor knowledge	115(41.2)	70(48.6)	Ref	Ref
Good knowledge	164(58.8)	74(51.4)	1.35(0.90–2.02)*	1.19(0.66–2.14)
Knowledge on reason for collecting and using aggregated age or sex of patient (or client) data				
Poor knowledge	93(33.3)	74(51.4)	Ref	Ref
Good knowledge	186(66.7)	70(48.6)	2.11(1.40–3.19)**	1.47(0.81–2.67)
Knowledge on reason for collecting and using aggregated geographical data				
Poor knowledge	38(13.6)	25(17.4)	Ref	
Good knowledge	241(86.4)	119(82.6)	1.33(0.77–2.31)	
Knowledge on why population data is needed				
Poor knowledge	63(22.6)	40(27.8)	Ref	Ref
Good knowledge	216(77.4)	104(72.2)	1.32(0.83–2.09)*	0.84(0.43–1.64)
Knowledge on dimensions of data quality				
Poor knowledge	37(13.3)	40(27.8)	Ref	Ref
Good knowledge	242(86.7)	104(72.2)	2.52(1.52–4.16)**	1.16(0.54–2.50)
Knowledge on data quality improving strategies				
Poor knowledge	94(33.7)	81(56.3)	Ref	Ref
Good knowledge	185(66.3)	63(43.7)	2.53(1.68–3.82)**	2.01(1.16–3.47)***
Competency of data analysis and interpretation				
Low competency	54(19.4)	92(63.9)	Ref	Ref
High competency	225(80.6)	52(36.1)	7.37(4.69–11.58)**	2.90(1.71–4.91)***
Organizational decision making climate				
Unfavorable	55(19.7)	84(58.3)	Ref	Ref
Favorable	224(80.3)	60(41.7)	5.70(3.66–8.89)**	2.61(1.43–4.77)***
Information use promotion by managers or supervisors				
Poor	63(22.6)	83(57.6)	Ref	Ref
Good	216(77.4)	61(42.4)	4.67(3.02–7.20)**	1.56(0.77–3.15)
Information use promotion by department staffs				
Unfavorable	43(15.4)	76(52.8)	Ref	Ref
Favorable	236(84.6)	68(47.2)	6.13(3.87–9.73)**	2.46(1.19–5.08)***
Health workers perception of data management				
Unfavorable	107(38.4)	75(52.1)	Ref	Ref
Favorable	172(61.6)	69(47.9)	1.75(1.16–2.62)**	0.84(0.44–1.62)
Health workers self-efficacy of data analysis, interpretation and use				
Low	30(10.8)	30(20.8)	Ref	Ref
High	249(89.2)	114(79.2)	2.18(1.26–3.80)**	2.51(1.17–5.36)***

RHI, routine health information; CI, confidence interval; Ref, reference category; COR, crude odds ratio; AOR, adjusted odds ratio

*P < 0.25 for COR, **P < 0.05 for COR, ***P < 0.05 for AOR

## Discussion

According to this study, two-thirds of health workers had good information usage practices. Two-thirds (67.1%) of health workers rated the organizational decision-making climate and information use promotion by facility managers or supervisors (65.5%) as favorable. The facility's information use promotion measures were rated positively by seven out of ten (71.9%) health workers.

Regarding RHIS data and its management, over half (57.0%) of health workers had a favorable perception. More than eight out of ten (85.8%) health workers believed that they had high self-efficacy in data analysis, interpretation, and use. However, only about two-thirds (65.5%) of health workers actually had high competency in data analysis and interpretation. Information utilization among health workers was predicted by service experience, title or position, work place, RHI training, knowledge of data quality, self-efficacy and competency in data analysis and interpretation, organizational decision making climate, and information promotion by department staff. The sexes had marginal associations with good information utilization and utilization.

Information use practices in the study area were comparable to a study done in Kenya (69.6%), East Wollega zone, Western Ethiopia (66%), and Hadiya zone, Southern Ethiopia (62.7%) [20, 25, 26]. This is better than studies done in the East Gojam zone, Northern Ethiopia (45.8%) [27] Diredawa, Eastern Ethiopia (53.1%) [28], Addis Ababa (37.3%) [18], Western Amhara (38.4%) [29], Oromia special zone (52.8%) [30], hospitals of Oromia regional state (56%) [31], and Southwest Ethiopia (57.3%) [32]. The finding was also better than a study conducted in selected districts of Amhara region (46%) [33], estimated pooled prevalence of information use at the national level (57.4%) [17], a study conducted in Tanzania [34] and another study conducted in Kenya among health care providers (34%) [35]. The current study's finding, on the other hand, was lower than that of a study conducted in North Gondar, Northwest Ethiopia (78.5%) [19], and the North Shewa zone of the Oromia region (71.6%) [36]. The possible explanation for the variations in study findings might be contextual differences, differences in the period of assessments, and scope of the study. However, the current level of information use at the point of data generation and supervisory level was unacceptably low in the study area. This has a considerable impact on the performance of the health system.

The majority of health workers have utilized health information to observe health service trends in their catchment area, identify and manage epidemics, drug supply and management, disease prioritization, and plan with an information utilization rate ranging from 72.8 to 81.8%. Only over half (56.3%) of health workers have utilized health data for day-to-day management of health services. That is a finding lower than a study conducted at health centers in Oromia special zone (77.5%) and public health centers in North Gondar (89.6%) [19, 30]. The good practice of information use by healthcare providers and managers helps to improving primary health care and achieve universal health coverage [37].

Two-thirds of health workers believe a favorable culture of health information use promotion exist in their organization, and managers or supervisors have a positive attitude towards information use. This finding was better than a study conducted in Southern Ethiopia in which 58.8% of health workers had a good perceived culture of health information [20] and that of Northern Ethiopia, where 48.1% of health workers had a good perceived culture of health information [19]. Besides, the majority of health workers had a favorable attitude towards data management, including data collection, organization, analysis, and reporting. It is believed that these organizational and behavioral factors enhance the proper utilization of health data in health institutions. Many barriers to information use are linked to organizational and behavioral factors, and as such strengthening routine information systems involves building an information culture where information is valued at all health system levels [10].

Though most health workers had high self-efficacy in data analysis and interpretation, the study revealed that only two-thirds of them were competent in data analysis and interpretation. In terms of RHI task competency, the findings of this study outperformed the findings of a study conducted in Northwest Ethiopia (East Gojam (51.5%) and North Gondar (29.9%) [19, 24], as well as a study conducted in Southern Ethiopia (Hadiya zone (56.7%) [20]. The variations in health workers’ RHI skills might be due to contextual differences, differences in the scope of the study, and differences in supports in terms of training and supervision. In comparison to other research regions, the majority of health workers in the study area have been trained (88.7%) and supervised (92.9%) with an emphasis on RHI tasks [19, 20, 27].

In the study area, proper information use practices were predicted by service experience, title or position held by health workers, trainings, the existence of a favorable organizational decision-making climate, information use practices by department staff, and self-efficacy and competency in data analysis and interpretation. In support of this, insufficient skill in information use core competencies, poor data quality, insufficient data availability, system design, relationships between actors who produce and use data, decision making autonomy and authority structures, information use leadership, information use culture, and low individual commitment and motivations are all barriers to information use in low and middle-income countries [38]. Similarly, another study revealed that awareness gaps, lack of motivating incentives, irregularity of supportive supervision, lack of community engagement in health report verification, and poor technical capacity of health professionals were found to be the major barriers to information use [39]. The presence of competent professionals in data analysis and interpretations remains a critical factor to improve information use practices at health care setting [40].

Proper information use practice was positively associated with health workers’ service experience, title or position possessed by health workers, and work place. Good health information utilization was four times more likely among health workers having service experience of greater than 10 years compared to those with less than 5 years. Moreover, health information utilization was more likely among head health workers than experts and health workers at admin units compared to health facilities. Experienced health workers had the knowledge and motivation to manage data and utilize information compared to less experienced ones as they felt more responsibility. Most health decisions are made by people in positions located at administrative units rather than health facilities.

In this study, information use practice was less likely among trained health workers compared to untrained health workers. This finding was in contradiction to other studies that found RHI training was positively associated with proper information utilization [17, 20, 27, 29, 30, 32]. In the study area, the majority of health workers were trained, with a higher proportion of them having received training before the 12 months of survey period and most had received RHI training with a component of data management and quality assurance. Training may be one factor influencing data management and information utilization, but so may changes in health workers' knowledge, attitude, motivation, and competency, and, in turn, information utilization may be influenced by supervision, mentorships and other interventions [41]. Besides coverage of RHI training, the of quality of delivery in terms of content, duration and frequency is critical to influence positive changes in information usage behavior [42].

Health workers’ knowledge of health data quality improving measures was positively associated with information use practices in that information use practice were two times more likely among health workers with good RHI knowledge. Health workers who have a better knowledge of data quality and its measures have a high probability of generating good quality data. Access to good quality data in turn influences better utilization of information. This finding was supported by other studies that showed health workers’ knowledge of RHI management was associated with good information utilization [17, 20].

Health workers’ self-efficacy in data analysis and interpretation was positively associated with good information use among health workers in that high self-efficacy health workers utilized information 2.5 times more than their counterparts. Likewise, health workers’ skill in data analysis and interpretation also showed a positive association, in which good information utilization was three times more likely among competent health workers in data analysis and interpretation. This finding was consistent with other studies [19, 20, 27, 30, 32]. Lack of skills to analyze, interpret, and use data among health workers impedes real-time decision making in organizations.

The existence of a favorable organizational decision-making climate and information promotion by department staff was positively associated with information use among health workers. Information use is about three times more likely among health workers’ in organizations with a favorable decision making climate and among health workers in organizations where information is promoted by department staff. Information use promotion culture in an organization is identified as an important factor in the effective utilization of information by its workers and managers. This finding was supported by other studies [18–20, 27]. The presence of regular supervision and managerial support, and provision of feedback is important to improve health workers’ commitment to information use [43] and enhance primary healthcare service delivery [44]. On the contrary, the social and political dynamics (such as political conflict, interest of significant others etc..) in decision making process hinders better information use practice [45].

In the bivariate analysis, variables such as sex of health workers, existence of supportive supervision, type of health institution, knowledge of collecting and using aggregated health data (disease, age and sex), and dimensions of data quality, and information use promotion by department managers or supervisors were shown to have an association. However, the association was not maintained when adjusted for other confounding variables. In other studies, these variables were statistically associated with good information utilization among health workers [17–20, 27–30, 32]. The organizational determinants, including feedback mechanisms, supportive supervision and resource availability in the health information system, were predictors of information use among health workers [46].

This study has assessed information use practices and associated factors guided by validated framework (PRISM framework) and by considering both organizational and individual factors comprehensively. Both healthcare providers and healthcare managers were included in the study. The assessment was conducted based on a representative district in the zonal administration and, hence, the findings have the possibility of generalizability. The study was not without limitations. Information use is measured based on the perception of health workers and this might obscure the actual practice. As the assessment was conducted at facility level, information bias might be introduced because the data collection setting is the same as the working environment. The health workers might be afraid to give the correct information. Since we have used data collectors outside of the study settings and adequate explanations were provided about the aim of the study to respondents, this bias might be minimized.

## Conclusions

Informed decision-making in primary health care (PHC) is a foundation of universal health coverage. This study concluded that about two thirds of health professionals practice proper information utilization, however the routine information usage at public health institutions remains lower than the regional and national expectations. This might impede efforts towards improving health system performance at the primary health care level. The study also revealed that health workers’ knowledge on health data quality, and their self-efficacy and skills of data analysis and interpretations, existence of information use promotion culture at organizations, among health care managers and department staffs were positively associated with information use practices among health workers. Therefore, efforts should be made to improve the organizational decision making climate, and health workers knowledge towards health data management and use. The value of data should be advocated and promoted at all levels of the health system.

## Supplementary Information

**Additional file 1.** Organizational and Behavioral Assessment Tool for Health Facility.

## Data Availability

The datasets used and/or analyzed during the current study are not openly available because data is part of ongoing research project and available from the corresponding author (dave86520@gmail.com or dawit.daka@ju.edu.et) on reasonable request.

## Abbreviations

AOR: Adjusted odds ratio
CI: Confidence interval
COR: Crude odds ratio
RHI: Routine health information
HIS: Health information system
HMIS: Health management information system
