# Supplementary material for: Health workers' use of routine health information and related factors at public health institutions in Illubabor Zone, Western Ethiopia
Source: BMC Med Inform Decis Mak. 2022 May 24;22:140. doi: 10.1186/s12911-022-01881-y (PMC9131521; doi:10.1186/s12911-022-01881-y)

# Health Facility Organizational Behaviral Assesment Tools (Health Facility Module)

Organizational and Behavioral Assessment Tool (OBAT)- For Health Facility

**125 Enter date of data collection**

yyyy-mm-dd

**126 Enter code for this questionnaire**

**127 Name of person filling this form**

Socio-demographic Information

## Sociodemographic Data of the Respondents

**128 Select your facility**

- ◯ Zonal health office
- ◯ Woreda health office
- ◯ Hospital
- ◯ Health center

**128.1 Woreda of the health institution**

*Select the Woreda in which the health institution is located.*

- ◯ Metu rural
- ◯ Metu town
- ◯ Becho
- ◯ Bilo Nopha
- ◯ Darimu
- ◯ Hurumu
- ◯ Yayo
- ◯ Alle

**128.2 Name of the health institution**

*Enter the name of the health institution as for example: Hurumu Woreda health office, Metu Karl Hospital, Hurumu health center, Illubabor zone health department etc..*

**129 Sex of the respondent**

- ◯ Male
- ◯ Female

**130 Age of the respondent**

**131 Title or Position of the respondent**

- ◯ Facility head
- ◯ Reform and plan head
- ◯ Department head
- ◯ Prevention coordinator
- ◯ Malaria, HIV/TB focal
- ◯ HEW coordinator
- ◯ HIT
- ◯ Other

**131.1 Other, specify**

**132 What type of formal technical/ Medical training did you attend?**

- ☐ MSc in M & E
- ☐ MSC in Health Informatics
- ☐ MPH or other related masters degree
- ☐ Physician
- ☐ Health officer
- ☐ Nurse /Midwife
- ☐ HIT /IT
- ☐ HEW
- ☐ Other

**132.1 Other, specify**

**133 Years of employment**
*year and then month*

______________________________

**134 Years of working with health data or RHIS/HMIS/CHIS**

______________________________

**135 Have you ever received formal RHIS/HMIS/CHIS training?**

- ○ Yes
- ○ No

**136 When did you receive the training?**

- ○ Yes, with in the past 12 month
- ○ Yes, before past 12 month

**137 What type of formal RHIS training have you ever received?**

- ☐ Health statistics
- ☐ HMIS /CHIS (Data collection, compilation, aggregation, transmission, storage, and /or data quality assurance)
- ☐ Data analysis and use
- ☐ General M&E
- ☐ ICT
- ☐ Other

**137.1 Other, specify**

______________________________

Information Use Culture

## We Would Like To Know Your Opinion (How Strongly You Agree Or Disagree) About Certain Aspects Of HIS use culture In Your Health Facility. The Scale Assesses the Intensity Of Your Belief And Ranges From Strongly Disagree (1) To Strongly Agree (5).

**138 In your health facility/Department decision are based on Personal preference of decision-makers.**

- Strongly disagree
- Disagree
- Neither agree nor disagree
- Agree
- Strongly agree

**139 In your health facility/Department decision are based on Superior directives.**

- Strongly disagree
- Disagree
- Neither agree nor disagree
- Agree
- Strongly agree

**140 In your health facility/Department decision are based on Evidence /Facts/ data.**

- Strongly disagree
- Disagree
- Neither agree nor disagree
- Agree
- Strongly agree

**141 In your health facility/Department decision are based on History, what was done last year?**

- Strongly disagree
- Disagree
- Neither agree nor disagree
- Agree
- Strongly agree

**142 In your health facility/Department decision are based on funding directives from higher levels.**

- Strongly disagree
- Disagree
- Neither agree nor disagree
- Agree
- Strongly agree

**143 In your health facility/Department decision are based on political considerations.**

- Strongly disagree
- Disagree
- Neither agree nor disagree
- Agree
- Strongly agree

**144 In your health facility/Department decision are based on official health sector strategic objectives.**

- Strongly disagree
- Disagree
- Neither agree nor Disagree
- Agree
- Strongly agree

**145 In your health facility/Department decision are based on health need of the catchment population as identified locally.**

- Strongly disagree
- Disagree
- Neither agree nor disagree
- Agree
- Strongly agree

**146 In your health facility/Department decision are based on the relative cost of interventions.**

- Strongly disagree
- Disagree
- Neither agree nor disagree
- Agree
- Strongly agree

**147 In your health facility/Department decision are based on participatory by taking inputs from relevant staffs.**

- Strongly disagree
- Disagree
- Neither agree nor disagree
- Agree
- Strongly agree

**148 Your health facility managers or supervisors Seek inputs from the relevant staffs.**

- Strongly disagree
- Disagree
- Neither agree nor dis agree
- Agree
- Strongly agree

**149 Your health facility managers or supervisors emphasis that data quality procedures be followed in the compilation and submission of the periodic report (Monthly report).**

- Strongly disagree
- Disagree
- Neither agree nor disagree
- Agree
- Strongly agree

**150 Your health facility managers or supervisors Promote feedback mechanism to share/present information within the team and to lower and upper levels of the system.**

- Strongly disagree
- Disagree
- Neither agree nor disagree
- Agree
- Strongly agree

**151 Your health facility managers or supervisors use RHIS data for service performance monitoring and target setting.**

- Strongly disagree
- Dis agree
- Neither agree nor disagree
- Agree
- Strongly agree

**152 Your health facility managers or supervisors emphasis the need to use RHIS data to identify potential disparities in service delivery or use.**

- Strongly disagree
- Disagree
- Neither agree Nor disagree
- Agree
- Strongly agree

**153 Your health facility managers or supervisors conduct routine data quality checks of points where data are captured, processed or aggregated.**

- Strongly disagree
- Disagree
- Neither agree nor disagree
- Agree
- Strongly agree

**154 Your health facility managers or supervisors ensure that performance data are reviewed and discussed in the regular meeting.**

- Strongly disagree
- Disagree
- Neither agree nor disagree
- Agree
- Strongly agree

**155 Your health facility managers or supervisors ensure that decision are made and follow-up action identified in PMT meetings based on presented data.**

- Strongly disagree
- Disagree
- Neither agree Nor disagree
- Agree
- Strongly agree

**156 Your health facility managers or supervisors provide regular feedback on reported data quality (E.g. accuracy data, compilation/reporting) to the staff responsible for compiling and reporting the data.**

- Strongly disagree
- Disagree
- Neither agree nor disagree
- Agree
- Strongly agree

**157 Your health facility managers or supervisors recognize or reward staff for good work performance**

- Strongly disagree
- Disagree
- Neither agree nor disagree
- Agree
- Strongly agree

**158 In the health department, staffs complete RHIS tasks (recording, reporting, processing, aggregation, and or analysis) on time.**

- Strongly disagree
- Disagree
- Neither agree nor disagree
- Agree
- Strongly agree

**159 In the health department, staffs display commitment to ensure data quality and evidence-based decision making**

- Strongly disagree
- Disagree
- Neither agree nor disagree
- Agree
- Strongly agree

**160 In the health department, staffs pursue indicative national targets and set the feasible local target for essential service performance**

- Strongly disagree
- Disagree
- Neither agree nor disagree
- Agree
- Strongly agree

**161 In the health department, staffs feel ``personal responsibility`` for failing to reach performance targets**

- Strongly disagree
- Disagree
- Neither agree nor disagree
- Agree
- Strongly agree

**162 In the health department, staffs use RHIS data for the day-to-day management of the facility and Woreda (E.g., service delivery, financial, commodities, and human resource management)**

- Strongly disagree
- Disagree
- Neither agree Nor disagree
- Agree
- Strongly agree

**163 In the health department, staffs use RHIS data to solve a common problem in service delivery**

- Strongly disagree
- Disagree
- Neither agree nor disagree
- Agree
- Strongly agree

**164 In the health department, staffs use disaggregated RHIS data to identify and/or solve health equity-related problem in service delivery**

- Strongly disagree
- Dis agree
- Neither agree nor disagree
- Agree
- Strongly agree

**165 In the health department, staffs prepare data visual (graph, tables, maps, etc.) showing achievement toward targets (indicators, geographic and/or temporal trends or situation data)**

- Strongly disagree
- Disagree
- Neither agree nor disagree
- Agree
- Strongly agree

**166 In the health department, staffs can monitor whether an initiative /intervention achieved the targets or goal can make decisions within their scope in response to the finding of data analysis (e.g. changes in service delivery or management practices**

- Strongly disagree
- Disagree
- Neither agree nor disagree
- Agree
- Strongly agree

**167 In the health department, staffs are held accountable for poor performance (e.g, failure to meet reporting deadlines)**

- Strongly disagree
- Disagree
- Neither disagree nor agree
- Agree
- Strongly agree

**168 In the health department, staffs admit mistakes (related to data management)if/when they occur and take corrective action**

- Strongly disagree
- Disagree
- Neither agree nor disagree
- Agree
- Strongly agree

Personal Feelings/ Opinions

______________________________

## The Scale Assesses the Intensity Of Your Belief And Ranges From Strongly Disagree (1) To Strongly Agree (5).

**169 I feel discouraged when the data that I collect/ record are not used for taking action/ either for monitoring or decision making**

- Strongly disagree
- Disagree
- Neither agree nor disagree
- Agree
- Strongly agree

**170 I find collecting /recording HMIS/CHIS data tedious**

- Strongly disagree
- Disagree
- Neither agree nor disagree
- Agree
- Strongly agree

**171 I collect data if meaningful (useful) for me**

- Strongly disagree
- Disagree
- Neither agree nor disagree
- Agree
- Strongly agree

**172 I feel that the data either I collect or I have are, important for monitoring or facility service performance**

- Strongly disagree
- Disagree
- Neither agree nor disagree
- Agree
- Strongly agree

**173 My work of collecting data is appreciated and valued by supervisors**

- Strongly disagree
- Disagree
- Neither agree Nor disagree
- Agree
- Strongly agree

**174 I feel that data collection/recording is not the responsibility of health care provider**

- Strongly disagree
- Disagree
- Neither agree nor disagree
- Agree
- Strongly agree

RHIS Knowledge

# Indicate Knowledge on Possible Reasons For Collecting or Using Aggregated Data On a Monthly Basis For The Following Data Types. (Circle Your Response Either Yes or No)

**175A What are the possible reason for collecting or using aggregated DISEASE data on a monthly or quarterly basis? (Select multiple)**

- [ ] To provide individual level care
- [ ] To know changes in magnitude/burden of selected diseases
- [ ] To plan preventive and promotive activities
- [ ] To refer patients/clients
- [ ] To identify disease outbreaks and take action to address epidemics
- [ ] No answer

**175B What are the possible reason for collecting or using aggregated IMMUNIZATION data on a monthly or quarterly basis? (Select multiple)**

- [ ] To know the coverage of effective intervention (immunization) for improving maternal or child health
- [ ] To improve diagnosis and treatment of under five children
- [ ] To take action for providing necessary resources (eg. staffing, equipment, vaccines, etc)
- [ ] To plan for immunization activities – developing targets for immunization
- [ ] To identify disease outbreaks and take action to address epidemics
- [ ] To punish parents who deny infant immunization
- [ ] No answer

**175C What are the possible reason for collecting or using aggregated 'Age/sex of patients/clients' data on a monthly or quarterly basis? (Select multiple)**

- [ ] To ensure equitable service coverage across people of all groups
- [ ] To improve the service delivery for women
- [ ] To know which group is affected by certain disease
- [ ] To know if the appropriate group is getting the relevant services
- [ ] To calculate workload of OPD and under-five clinic
- [ ] No anwer

**175D What are the possible reason for collecting or using aggregated 'Geographical data or residence of patients, i.e., where they come from' data on a monthly or quarterly basis? (Select multiple)**

- [ ] To plan preventive and promotive activities targeted to certain geographic areas
- [ ] To know their economic status of the population
- [ ] To improve access and utilization of health services
- [ ] To determine the behavior of clients/population group
- [ ] For disease surveillance (to control epidemic/disease outbreaks)
- [ ] No answer

**175E Why are population data needed (e.g., number of people living in the catchment area, ...)? (Select multiple)**

- [ ] To use as denominator for calculating of indicators
- [ ] To plan the offer of various health services
- [ ] To calculate the workload of health facilities
- [ ] To know the knowledge and skill of health professionals
- [ ] No answer

**175F Describe at least three aspects OR DIMENSIONS of data quality. (select multiple)**

- [ ] Data accuracy or precision
- [ ] Report timeliness
- [ ] Report/data Completeness
- [ ] Reliability
- [ ] Consistency
- [ ] No answer

**175G Describe at least three ways of ensuring the data quality relevant to your job classification/responsibilities.**

- [ ] Observation of the service provider for correct diagnosis and documentation
- [ ] Cross check recorded data against reported data (recount data from source document and compare it with the reported data)/ LQAS
- [ ] Review records or reports and identify data entry problems or errors
- [ ] Use in-built electronic data validation rules to review data quality
- [ ] Internal consistency – e.g. comparison of number of patient and amount of drug dispensed
- [ ] External consistency - comparison of indicator calculated from routine data with the same indicator calculated using data from other sources
- [ ] Historical comparison
- [ ] Having standard definitions of indicators and data elements
- [ ] Follow standardized data collection and data sources(registers, tally sheet, etc)
- [ ] Follow standardized guideline on data recording, reporting and data quality checks
- [ ] Train health workers and HITs on the standard HMIS guidelines
- [ ] Mentorship and supervision on data quality, recording and reporting
- [ ] no answer

Supervision and Mentor

______________________________

## This Part Contain Question Which Shows That Supervision and Mentorship Status On RHIS Data Utilization

**180 Do the supervisors visit your organization?**

- ○ Yes
- ○ No

**181 Do the supervisors mentor you?**

- ○ Yes
- ○ No

**182 How many times do they visit you in the last 6 months?**

- ◯ no visit
- ◯ 1 times
- ◯ 2 times
- ◯ 3 times
- ◯ 4 times
- ◯ 5 times
- ◯ 6 times

**183 From where the supervisors come? [SELECT MULTIPLE]**

- ☐ Federal Ministry of Health
- ☐ Regional health bureau
- ☐ Zonal health office
- ☐ Woreda Health office
- ☐ Donor or partner
- ☐ Other

**183.1 Other, specify**

---

Information Use Practice

---

## This Part Used To Assess Information Use Practical and The Scale Assesses the Intensity Of Your Belief And Ranges From Strongly Disagree (1) To Strongly Agree (5).

**184 I often use data for the day-to-day management of health service**

- ◯ Strongly disagree
- ◯ Disagree
- ◯ Neither agree nor disagree
- ◯ Agree
- ◯ Strongly agree

**185 I often use data to identify and manage epidemics**

- Strongly disagree
- Disagree
- Neither agree nor disagree
- Agree
- Strongly agree

**186 I use data to observe the trend of health services in my catchment**

- Strongly disagree
- Disagree
- Neither agree nor disagree
- Agree
- Strongly agree

**187 I often use data for planning**

- Strongly disagree
- Disagree
- Neither agree nor disagree
- Agree
- Strongly agree

**188 I use data for drug supply and management**

- Strongly disagree
- Disagree
- Neither agree nor disagree
- Agree
- Strongly agree

**189 I often use data for disease prioritization**

- Strongly disagree
- Disagree
- Neither agree nor disagree
- Agree
- Strongly agree

**190 I often use data for resource allocation**

- Strongly disagree
- Disagree
- Neither agree nor disagree
- Agree
- Strongly agree

**191 I use data for monitoring staffs performance**

- Strongly disagree
- Disagree
- Neither agree nor disagree
- Agree
- Strongly agree

**192 I use data for decision making**

- Strongly disagree
- Disagree
- Neither agree nor disagree
- Agree
- Strongly agree

**193 I often use data for community mobilization and discussion**

- Strongly disagree
- Disagree
- Neither agree nor disagree
- Agree
- Strongly agree

## SELF PERCEPTION OF COMPETENCY TO PERFORM RHIS TASKS.

We are interested in knowing how competent you feel in performing RHIS-related tasks. Please be frank and rate your competence honestly.

*Please rate your competence to accomplish the various RHIS activities on a scale from 0-10, where 0 is no competency and 10 is best.*

**194 I can check data accuracy**

*Please rate your competence to accomplish the various RHIS activities on a scale from 0-10, where 0 is no competency and 10 is best.*

- 1
- 2
- 3
- 4
- 5
- 6
- 7
- 8
- 9
- 10

**195 I can calculate percentages/rates correctly**

*Please rate your competence to accomplish the various RHIS activities on a scale from 0-10, where 0 is no competency and 10 is best.*

- 1
- 2
- 3
- 4
- 5
- 6
- 7
- 8
- 9
- 10

**196 I can plot a trend on a chart**

*Please rate your competence to accomplish the various RHIS activities on a scale from 0-10, where 0 is no competency and 10 is best.*

- ○ 1
- ○ 2
- ○ 3
- ○ 4
- ○ 5
- ○ 6
- ○ 7
- ○ 8
- ○ 9
- ○ 10

**197 I can explain the findings of the data analysis and their implications**

*Please rate your competence to accomplish the various RHIS activities on a scale from 0-10, where 0 is no competency and 10 is best.*

- ○ 1
- ○ 2
- ○ 3
- ○ 4
- ○ 5
- ○ 6
- ○ 7
- ○ 8
- ○ 9
- ○ 10

**198 I can use data for identifying performance gaps (e.g., service, program, managerial, …) and its root cause**

*Please rate your competence to accomplish the various RHIS activities on a scale from 0-10, where 0 is no competency and 10 is best.*

- 1
- 2
- 3
- 4
- 5
- 6
- 7
- 8
- 9
- 10

**200 I can use data for making operational/ management decisions, e.g., for service delivery, setting performance targets budget allocation, distribution of roles and responsibilities, staff assignment, logistics distribution.**

*Please rate your competence to accomplish the various RHIS activities on a scale from 0-10, where 0 is no competency and 10 is best.*

- 1
- 2
- 3
- 4
- 5
- 6
- 7
- 8
- 9
- 10

# This Part Used To Analyze the Skill Level of The Respondent. Please Collect the Data On Paper Based Format First. do not for gate: To Submit the Remaining Hard Copy For the Facilitators.

**201.1 Describe the data quality problem in this scenario**

*Each correct answer gets one point with a maximum score of 2 points (one for each criteria). If incorrect, the score is zero.*

- 1 (for 1 correct answer)
- 2 (for 2 correct answer )
- 0 ( incorrect answer)

**201.2 Potential reasons to the data quality problem**

*Each correct answer gets one point with a maximum score of 3 points. Wrong answers (or no answers) get a score of zero. The range would vary between 0 and 3.*

- 1 (1 correct answer given)
- 2 (2 correct answer given )
- 3 (3 correct answer given)
- 0 (no correct answer is given)

**201.3 Major activities to improve the data quality**

*Each correct answer gets one point with a maximum score of 5 points. Wrong answers (or no answers) get a score of zero. The overall raw score is obtained by adding all the scores. The range would vary between 0 and 5*

- 1 (1 correct answer is given)
- 2 (2 correct answer is given)
- 3 (3 correct answer is given)
- 4 (4 correct answer is given)
- 5 (5 correct answer is given)
- 0 (no correct answer is given)

**202 The estimated number of pregnant mothers in the facility catchment area for the current period is 340. The antenatal clinic in your facility has registered 170 pregnant mothers. Calculate the percentage of pregnant mothers in the facility catchment area attending antenatal care.**

*A correct answer gets one point. Wrong answers (or no answers) get a score of zero*

- 1
- 0

**203.1 Develop a line graph depicting the trend over one year in Iron 90+ coverage among women attending ANC for the first time.**

*Correct presentation of the line graph gets one point. Wrong answers (or no answers) get a score of zero*

- 1
- 0

**203.2 What does the following graph tell you about the FP method mix for new users in health center Y?**

*Each correct answer gets one point. Wrong answers (or no answers) get a score of zero. The overall raw score is obtained by adding all the scores. The range would vary between 0 and 2*

- 1 (1 answer is given)
- 2 (2 answer is given)
- 0 (no answer is given)

**203.3 The target for health center Y for new clients on FP for the year 2008 is 1,200 clients. How many new clients would the facility need to have each month if new clients were evenly distributed by month?**

*each correct answer gets one point. Wrong answers (or no answers) get a score of zero. The overall raw score is obtained by adding all the scores. The range would vary between 0 and 2.*

- ◯ 1 (if 1 answer is given)
- ◯ 0 (no correct answer is given)

**204.1 Provide at least one use of above graph findings at Facility level:**

*One or more correct answer(s) gets one point. Wrong answers (or no answers) get a score of zero. The range would vary between 0 and 1.*

- ◯ 1
- ◯ 0

**204.2 Provide at least one use of above graph findings at Community level:**

*One or more correct answer(s) gets one point. Wrong answers (or no answers) get a score of zero. The range would vary between 0 and 1.*

- ◯ 1
- ◯ 0

**205.1 What is the malnutrition rate of boys?**

*A correct answer gets one point. Wrong answers (or no answers) get a score of zero.*

- ◯ 1
- ◯ 0

**205.2 What is the malnutrition rate of girls?**

*Scoring: A correct answer gets one point. Wrong answers (or no answers) get a score of zero*

- ◯ 1 (if one point is listed)
- ◯ 2 (if two point is listed)
- ◯ 0 (if no any point is listed)

**205.3 What information do you get by disaggregating the data by sex? How does this information help you in planning/improving your service delivery?**

*Each correct answer gets one point with a maximum score of 2 points. Wrong answers (or no answers) get a score of zero. The range would vary between 0 and 2.*

- ◯ 1 (if one point is listed)
- ◯ 2 (if two point is listed)
- ◯ 0 (if no any point is listed)

**Record your current location**

latitude (x.y °)

longitude (x.y °)

altitude (m)

accuracy (m)

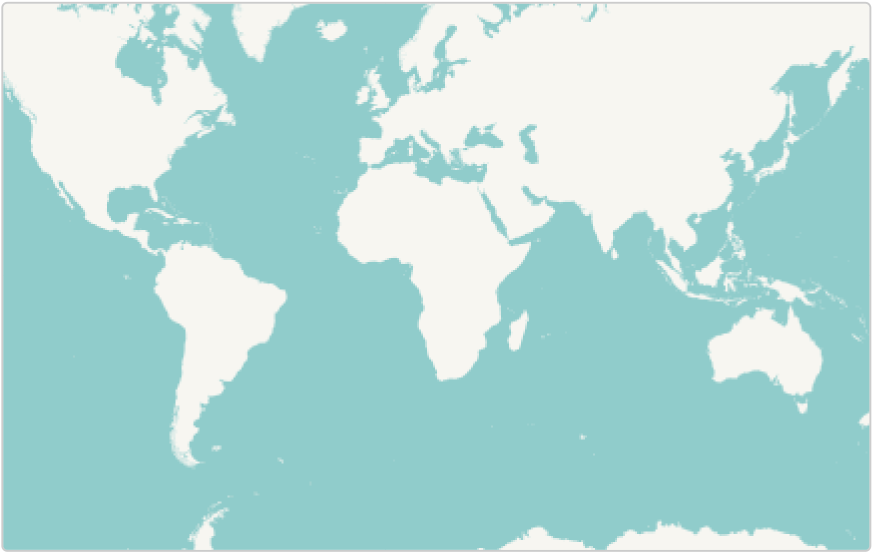

Supplement: Supplementary file 1 — Additional file 1. Organizational and Behavioral Assessment Tool for Health Facility. [file 12911_2022_1881_MOESM1_ESM.pdf]
